# Combined supplementation of essential oils, *Saccharomyces cerevisiae* and isomaltooligosaccharides improves intestinal absorption and immune functions in weaned piglets

**DOI:** 10.1093/jas/skaf391

**Published:** 2025-11-09

**Authors:** Luwei Zhu, Xiaohan Zhang, Zhiheng Yang, Zitao Fan, Chengyong Lv, Jianxing Qiu, Tianfang Xiao, Dingcheng Ye, Pingting Guo

**Affiliations:** College of Animal Science, Fujian Agriculture and Forestry University, Fuzhou, 350002, China; College of Animal Science, Fujian Agriculture and Forestry University, Fuzhou, 350002, China; College of Animal Science, Fujian Agriculture and Forestry University, Fuzhou, 350002, China; College of Animal Science, Fujian Agriculture and Forestry University, Fuzhou, 350002, China; College of Animal Science, Fujian Agriculture and Forestry University, Fuzhou, 350002, China; College of Animal Science, Fujian Agriculture and Forestry University, Fuzhou, 350002, China; College of Animal Science, Fujian Agriculture and Forestry University, Fuzhou, 350002, China; Institute of Animal Husbandry and Veterinary Medicine, Fujian Academy of Agricultural Sciences, Fuzhou, 350013, China; College of Animal Science, Fujian Agriculture and Forestry University, Fuzhou, 350002, China; Engineering Research Center for Animal Breeding and Sustainable Production, College of Animal Sciences, Fujian Agriculture and Forestry University, Fuzhou, 350002, China

**Keywords:** Yeast, cinnamaldehyde, thymol, oligosaccharide, intestine function, immunity

## Abstract

This study evaluated the synergistic effects of essential oils (EOs), *Saccharomyces cerevisiae* (SC), and isomaltooligosaccharides (IMO) on intestinal health and systemic immunity in weaned piglets using a randomized block design across two commercial farms. Within each farm, 108 piglets weaned at 24 d of age were randomly divided into 3 groups (3 pens per group, 12 piglets per pen). The piglets were fed a basal diet (CON) and a basal diet supplemented with antibiotics (0.5 kg/t colistin sulfate + 0.5 kg/t tilmicosin) (ANTI) or additive mixture (0.1 kg/t EOs, 0.3 kg/t SC and 1 kg/t IMO) (ESI) for 28 days. The results showed that both ESI and ANTI treatments significantly increased the ratio of villus height to crypt depth (*P *< 0.05). Specifically, ESI supplementation elevated serum IgA, hepatic IgA, and jejunal mucosal sIgA levels (*P *< 0.05), while upregulating hepatic *IL1B*, *IL2*, *IL6*, and *IFNG* expression alongside jejunal mucosal *IL1B*, *IL6*, and *PPARG* expression—contrasting with its downregulation of jejunal mucosal *IFNG* mRNA (*P *< 0.05). Conversely, compared with the CON group, ANTI supplementation increased serum IgA level and jejunal mucosal concentrations of sIgA, IgG, IgM, IFN-γ, and IL-10, while also upregulating jejunal mucosal gene expression of *IGF1* and *TGFB1* (*P *< 0.05). In addition, compared with Farm I, piglets at Farm II exhibited higher jejunal pH, elevated serum levels of IgA and IL-1β, and upregulated gene expression of *IL1B*, *IL2*, *IL6*, and *IFNG* in the liver as well as *TGFB1*, *IL1B*, *IL6*, and *PPARG* in the jejunal mucosa (*P *< 0.05). Moreover, the robust diet × farm interaction effects on immune gene expression in the liver (*IL1B*, *IL2*, and *IL6*) and jejunal mucosa (*IL1B*, *IL2*, *IL6*, *IFNG*, *IL10*, and *PPARG*) were observed (*P *< 0.05). Collectively, ESI improved jejunal epithelial maturation, nutrient absorption, and immune function in weaned piglets, with efficacy partly dependent on farm environmental conditions.

## Teaser

This multi-farm intervention study reveals that a natural feed blend (essential oils, *Saccharomyces cerevisiae*, and isomaltooligosaccharides) can enhance intestinal health and immunity in weaned piglets, though its effectiveness depends on farm-specific environmental conditions.

## Introduction

In modern intensive swine production systems, early weaning technology has been widely adopted by large-scale commercial farms to enhance sow productivity and economic efficiency. However, the sudden separation from the sow, combined with the abrupt loss of maternal antibody protection, gastrointestinal tract immaturity, and environmental challenges such as transportation and social regrouping, collectively trigger weaning stress in piglets (Spreeuwenberg et al., 2001; Campbell et al., 2013; Upadhaya and Kim, 2021). This stress manifests as a range of physiological disruptions, including gut microbiota dysbiosis, elevated oxidative stress, impaired intestinal epithelial integrity, and compromised digestive and barrier functions, ultimately impairing piglet growth and health (Wijtten et al., 2011; Moeser et al., 2017; Guevarra et al., 2019; Tang et al., 2022). Although antibiotics were historically used to mitigate post-weaning issues such as diarrhea, growing concerns over antibiotic resistance and residues have led to strict regulatory bans—such as those implemented in the European Union and China—intensifying the need for safe and effective non-antibiotic alternatives. In this context, essential oils (EOs), *Saccharomyces cerevisiae* (SC), and isomaltooligosaccharides (IMO) have emerged as promising candidates.

EOs are natural, highly concentrated aromatic oils extracted from the seeds, fruit peels, roots, stems, and leaves of aromatic plants through methods such as distillation, microwave extraction, and cold pressing (Rahim et al., 2023). Research showed that known EOs, including carvacrol, thymol, cassia bark-aldehyde, and menthol, could exert antibacterial and antioxidant effects through various mechanisms (Tariq et al., 2019; Diniz do Nascimento et al., 2020; Álvarez-Martínez et al., 2021). Studies also found that combining different EO was more effective than using them individually. For example, dietary supplementation with 5% cassia bark-aldehyde, 5% carvacrol, and 0.8% thymol enhanced the intestinal absorption area, improved barrier integrity, and increased digestive enzyme activity in weaned piglets, thereby boosting production performance (Zhao et al., 2023). Furthermore, carvacrol and thymol supplementation reduced intestinal inflammation and increased villus length in this cohort (Peng et al., 2022). Additionally, blended essential oils (containing 2.64% carvacrol, 1.34% thymol, and 13.80% cinnamaldehyde) improved ileal epithelial development and mitigated weaning stress in early-weaned piglets (Shao et al., 2023). SC, also known as brewer’s yeast or bread yeast, a single-celled eukaryotic organism, was reported to enhance immune function, improve growth performance, and inhibit the colonization of pathogenic bacteria (Elghandour et al., 2020). This immunomodulatory effect is driven by direct interactions between yeast cell wall components and host immune cells. As a major structural polysaccharide in the SC cell wall, mannan elicits innate and adaptive immune responses through augmentation of humoral and cellular immunity (Jin et al., 2019). IMO is a sweet white powder extracted from wheat, barley, potatoes, cassava, maltose, sucrose, and dextran (Tanriseven and Gokmen, 1999; Lee et al., 2002; Zhang et al., 2009). It is a disaccharide composed of two glucose molecules linked by an α-2,6 glycosidic bond and is classified as a functional oligosaccharide difficult to break down and absorb through digestive enzymes. Instead, IMO is utilized by hindgut microbes (*Bifidobacterium*, *Lactobacillus*, *Bacteroides* and so on) and stimulates the growth and reproduction of these probiotics (Kohmoto et al., 1984; Yen et al., 2011).

While EOs, SC, and IMO have each demonstrated efficacy in alleviating specific aspects of weaning stress—such as EOs against oxidative stress and pathogens, SC in enhancing immune and barrier functions, and IMO in modulating gut microbiota—their individual applications only partially address the multifaceted challenges of weaning stress. To achieve a more comprehensive mitigation of weaning stress, we hypothesize that a ternary combination (designated ESI) can integrate their complementary strengths: EOs suppressing pathogens and oxidative stress, SC enhancing immune and epithelial resilience, and IMO reshaping a healthy microbiota structure. Additionally, given the variable efficacy of many antibiotic alternatives across different farming systems, this study aims to systematically evaluate the effects of the ESI combination under varied commercial conditions. Our objective is to assess its role in regulating intestinal digestion-absorption and immune functions in weaned piglets, thereby providing a mechanistic basis for its application as an effective strategy to alleviate weaning stress and replace antibiotics.

## Material and Methods

The protocol for the present experiment was approved by the Animal Care and Use Committee of Fujian Agriculture and Forestry University.

### Experimental materials

EOs (15% thymol and 15% cinnamaldehyde) used in the present study, Anhuihua, were obtained from DuPont Nutrition & Health Animal. SC (*Saccharomyces cerevisiae*, 10^10^ CFU/g) was provided by Shandong Yihao Biotechnology Co., Ltd (Qingdao, China). IMO (90% content) was supplied by Shandong Tianmei Biotechnology Co., Ltd (Heze, China). Feed raw materials were obtained from Fujian Aonong Biological Technology Group Co., Ltd (Zhangzhou, China).

### Experimental design and feeding management

The experiment adopted a randomized complete block design with two commercial farms in Fujian Province, China (Farm I and Farm II) serving as blocks. Farm I operated with mechanical mixing, warm-air heating, and automated manure removal, while Farm II operated with manual mixing, heat-lamp heating, and manual manure removal. Within each farm, 108 healthy 24-day-old weaned piglets (Duroc × Landrace × Yorkshire) with an initial body weight (BW) of 7.59 ± 0.13 kg were randomly assigned to three dietary treatment groups. Each group comprised three replicates of 12 piglets per replicate (half male and half female), ensuring randomization across both farms to control location-specific variability. The 28-day trial included two feeding phases: days 1–14 and days 14–28 post-weaning. Dietary treatment groups included (1) basal diet (CON), (2) basal diet supplemented with antibiotics (0.5 kg/t colistin sulfate + 0.5 kg/t tilmicosin) (ANTI), and (3) basal diet supplemented with additive mixture (0.1 kg/t EOs, 0.3 kg/t SC and 1 kg/t IMO) (ESI). The basal diet was formulated according to the Chinese Nutrient Requirements of Swine (GB/T 39235-2020), with its composition and nutritional levels detailed in Table 1. Antibiotics and additives were administered at the manufacturer-recommended dosage. Pigs were fed twice daily (08:00 and 16:00) with ad libitum access to feed and water. All piglets were vaccinated post-weaning as per protocol.

**Table 1.** Composition and nutrient levels of basal diets (air-dry basis, %)

Items	Days 1–14	Days 15–28
**Ingredients**		
**Corn**	33.34	43.37
**Extruded corn**	30.00	25.00
**Soybean meal (46% CP)**	4.00	5.00
**Fish meal**	4.00	3.00
**Fermented soybean meal**	4.00	5.00
**Extruded soybean**	10.00	8.00
**Soybean oil**	1.00	0.00
**Soybean protein concentrate**	5.00	3.00
**Whey powder (3% CP)**	5.00	4.00
***L*-Lysine hydrochloride (98.5%)**	0.25	0.20
***DL*-Methionine**	0.05	0.03
***L*-Threonine**	0.18	0.14
**Tryptophan**	0.10	0.08
**Choline chloride (50%)**	0.08	0.08
**Zinc oxide**	0.20	0.20
**Dicalcium phosphate**	1.00	1.00
**Limestone**	0.60	0.70
**Sodium chloride**	0.20	0.20
**1% premix^1^**	1.00	1.00
**Total**	100.00	100.00
**Nutrient levels^2^**		
**Digestive energy (MJ/kg)**	14.53	14.23
**Crude protein**	18.83	17.36
**Ether extract**	5.62	4.36
**Crude fiber**	1.82	1.75
**Calcium**	0.73	0.72
**Available phosphorus**	0.40	0.38

1 The premix provides following per kilogram of the diet: vitamin A 12,000 IU; vitamin D_3_ 2,500 IU; vitamin E 30 IU; vitamin K_3_ 3 mg; vitamin B_1_ 1.5 mg; riboflavin 4 mg; niacin 40 mg; pantothenic acid 15 mg; vitamin B_6_ 3 mg; biotin 0.1 mg; vitamin B_12_ 0.012 mg; choline chloride 400 mg; folic acid 0.7 mg; Zn 100 mg; Mn 40 mg; Fe 90 mg; Cu 200 mg; I 0.35 mg; Se 0.3 mg.

2 Nutrient levels were calculated.

### Sampling

On d 28, one healthy weaned piglet per replicate was randomly selected for bleeding via the precaval vein. Blood samples were collected into 15 mL blood collection tubes, followed by 3,500 × rpm at 4 °C for 10 min, and the serum samples were frozen at −80°C for subsequent analyses. Afterwards, pigs were anaesthetized with sodium pentobarbital (50 mg/kg BW) and euthanized. A piece of liver was cut from the left lobe. The jejunum was sectioned at the middle and digesta was also taken. Approximately 2 cm jejunum segment was collected for histological analyses. At the same time, jejunal mucosa was scraped by a sterile glass microscope slide at 4 °C. Then, all samples were rapidly frozen in liquid nitrogen and stored at −80°C until analyses.

### Jejunal digestive enzyme activities

Approximately 0.1 g of frozen jejunal mucosa samples were homogenized in Phosphate-Buffered Saline (1:9, wt/vol) and then centrifuged at 3,000 × g for 20 min at 4 °C. The supernatant was used to determine the trypsin, lipase and amylase activities. All kits (trypsin, A080-2-2; lipase, A054-2-1; amylase, C016-2-1) were purchased from Nanjing Jiancheng Bioengineering Institute (Jiangsu, China), and the assays were carried out in accordance with instructions. Trypsin activity was determined by ultraviolet (UV) spectrophotometry. Absorbance (OD) was measured at 253 nm using a UV spectrophotometer (UV-1800PC; MAPADA, Shanghai, China). Lipase activity was determined using the microplate method with the OD recorded at 580 nm; meanwhile, amylase activity was determined using the colorimetry with the OD recorded at 540 nm.

### Jejunal pH and ORP measurement

A 1 g aliquot of the jejunal chyme sample was dissolved in 10 mL distilled water to prepare the test solution. The prepared solution was centrifuged at 8,000 × rpm for 10 min. The supernatant was then collected, and pH and oxidation-reduction potential (ORP) were measured using a pre-calibrated pH meter PHS-3E from Shanghai INESA Scientific Instrument Co., Ltd (Shanghai, China).

### Jejunal morphometric analysis

After slaughter, the mid-jejunum samples were quickly fixed in fixative solution. After 24 h of standing, the samples were removed from fixative solution, then dehydrated, and embedded in paraffin wax. Subsequently, each sample was sectioned at 5 μm thickness and stained with hematoxylin and eosin (H&E). Following staining, tissue sections underwent sequential dehydration through three changes of absolute ethanol (2 min each), followed by two changes of n-butanol (2 min each) and subsequent clearing in two changes of xylene (2 min each). Then, sections were mounted with neutral balsam under coverslips. Finally, the villus height to crypt depth (VH/CD) ratio was calculated by examining ten randomly selected villi height (VH) and crypt depth (CD) per histological section under a Motic BA210 Digital upright fluorescence microscope (Motic, Fujian, China).

### Development-related gene expression analysis in jejunal mucosa

Reference previous research methods (Wu et al., 2024), the expressions of development-related genes in jejunal mucosa, including *GCG*, *IGF1*, *IGF1R*, *TGFB1*, and *TGFB3* genes in the jejunum mucosa were detected by quantitative Reverse Transcription Polymerase Chain Reaction (qRT-PCR) analysis. Initially, the TRIzol reagent (TaKaRa, Dalian, China) was employed to obtain the total RNA from jejunal mucosa. Subsequently, the purity and quality of total RNA were identified spectrophotometrically via usage of OD260 and OD280 measurements (UV-1800PC; MAPADA, Shanghai, China). Following quality verification, total RNA was reverse-transcribed using the Evo M-MLV RT Mix Kit with gDNA Clean for qPCR Ver.2 (AG11728; Ruizhen Biotechnology Co., Ltd Guangzhou China) following the manufacturer’s protocol. Specifically**, t**he reaction system of 20 μL comprised 10 μL qPCR SYBR Green Master Mix (2×) (11201ES08; Yeasen Biotechnology Co., Ltd Shanghai, China), 1.5 μL cDNA templates, 0.4 μL of each primer (10 μM), and 7.7 μL of RNA enzyme-free water. The PCR program was guided as follows: 95 °C for 2 min, 40 cycles of 95 °C for 10 s, and 60 °C for 30 s. To normalize gene expression, the *GAPDH* gene was chosen as the internal reference. Finally, the relative expression of target genes was calculated using the 2^−ΔΔCt^ method. Additionally, the primer sequences of housekeeping and target genes were showed in Table 2.

**Table 2.** Primer sequences used for quantitative RT-PCR

Gene	Primer sequences (5′→ 3′)	Accession No.	Product length, bp
**Development-related genes**			
***GAPDH***	F: GTTCCAGTATGATTCCACCCAC R: TTCACGCCCATCACAAACAT	NM_001206359.1	270
***IGF1***	F: CTGAGGAGGCTGGAGATGTACT R: CCTGAACTCCCTCTACTTGTGTTC	NM_214256.1	137
***IGF1R***	F: TTCGCCAGATCCTAGGGGAG R: TCCCAGCTTTGATGGTCAGG	NM_214172.1	120
***GCG***	F: ACTCACAGGGCACGTTTACCA R: AGGTCCCTTCAGCATGTCTCT	NM_214324.1	150
***TGFB1***	F: TGACCCGCAGAGAGGCTAT R: CGGCCAGAATTGAACCCGT	NM_214015.2	106
***TGFB3***	F: GGCTGGAGGAGCACAATGAT R: CACTGACGACACGTTGAAGC	NM_214198.1	114
**Immune-related genes**			
***RPL4***	F: TGAGCTCTATGGCACTTGGC R: CGATGAATCTTCTTGCGTGGTG	NC_010443.5	150
***IL1B***	F: CAGCCAGTCTTCATTGTTCAG R: GTTTTGGGTGCAGCACTTCAT	NM_214055.1	150
***IL2***	F: TCAACTCCTGCCACAATGTATAAGA R: CTTGAAGTAGGTGCACCGTTTG	XM_021100436.1	89
***IL6***	F: CAGACCCTGAGGCAAAAGGGAA R: CTCAGGTGCCCCAGCTACAT	NM_214399.1	200
***IL10***	F: CTGCCCTGTGAAAACAAGAGC R: CCCTCTCTTGGAGCTTGCTAA	NM_214041.1	70
***PPARG***	F: GCCGTGTCTGTGGGGATAAA R: GCCCAAACCTGATGGCGTTA	NM_214379.1	219
***IFNG***	F: CGCAAAGCCATCAGTGAACTCATC R: TTTGATGCTCTCTGGCCTTGGAAC	NM_213948.1	110

### Immunoglobulin and cytokine quantification

Enzyme-linked immunosorbent assay was performed to quantify immunoglobulin A (IgA) (ml026837), immunoglobulin G (IgG) (ml002328), immunoglobulin M (IgM) (ml002334), interleukin-1β (IL-1β) (ml025973), interleukin-2 (IL-2) (ml025973), interleukin-6 (IL-6) (ml025981), interleukin-10 (IL-10) (ml025956), tumor necrosis factor-α (TNF-α) (ml002360), and interferon gamma (IFN-γ) (ml002333) concentrations in serum, hepatic tissue, and jejunal mucosa, with secretory immunoglobulin A (sIgA) (ml026686) specifically measured in jejunal mucosa. All assays were conducted in strict accordance with manufacturer-specified protocols (Shanghai Enzyme-linked Biotechnology Co., Ltd Shanghai, China).

### Immune-related gene expression analysis

Reference previous research methods (Wu et al., 2024), the expressions of immune-related genes, including *IL1B*, *IL2*, *IL6*, *IL10*, *PPARG* and *IFNG* genes in the liver and jejunum mucosa were detected by qRT-PCR analysis. Initially, total RNA was extracted from tissue samples using the TRIzol reagent (TaKaRa, Dalian, China) and purity was spectrophotometrically quantified. Subsequently, total RNA was reverse-transcribed using the Evo M-MLV RT Mix Kit with gDNA Clean for qPCR Ver.2 (AG11706; Accurate Biotechnology Co., Ltd Hunan, China). qRT-PCR of these genes was carried out using the same method established for the development-related genes. The relative expressions of target genes were calculated using the 2^−ΔΔCt^ method with *RPL4* gene as the internal reference. Primer sequences are provided in Table 2.

### Statistical analysis

Statistical analysis was performed using SPSS 26.0 software (IBM Corp., Armonk, NY, USA). Data were analyzed using ANOVA with the following linear model:


$$Y=\mu +F_{i}+T_{j}+{\left(F\times T\right)}_{\left\{ij\right\}}+\varepsilon_{\left\{\mathrm{ijk}\right\}}$$

where Y is the dependent variable, μ is the overall mean, F_i_ is the fixed effect of farm (i = I, II), T_j_ is the fixed effect of dietary treatment (j = CON, ANTI, ESI), (F × T)_{ij}_ is the interaction effect between farm and dietary treatment, and ε_{ijk}_ is the residual error. When a significant interaction (*P *< 0.05) was detected, simple effects were examined; otherwise, the main effects of farm and dietary treatment were interpreted. For any significant main effects, Duncan’s multiple range test was employed for post hoc mean comparisons. Statistical significance was set at *P *< 0.05, while highly significant differences were defined as *P *< 0.01. All data are shown as the mean and standard error of mean (SEM).

## Results

### Activities of jejunal digestive enzymes

**Figure 1.**
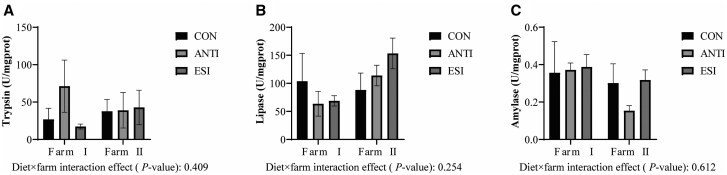
Effects of ESI on jejunal digestive enzyme activity in weaned piglets across farm environments (U mg protein^−1^): trypsin activity (A), lipase activity (B), amylase activity (C). CON, basal diet; ANTI, basal diet + antibiotics; ESI, basal diet + EOs + SC + IMO. EOs, essential oils. SC, *Saccharomyces cerevisiae*. IMO, isomaltooligosaccharides. Data are presented as mean ± SEM.

The changes in jejunal mucosal digestive enzyme activities (lipase, amylase and trypsin) are presented in Figure 1. No significant differences were observed in jejunal digestive enzyme activities across dietary treatments, nor was there any effect of farm environment on these enzymatic activities (*P *> 0.05).

### Jejunal pH and ORP measurement

**Figure 2.**
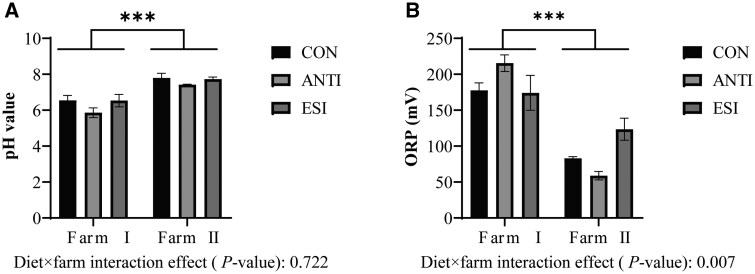
Effects of ESI on jejunal chyme pH (A) and ORP (B) in weaned piglets across farm environments. CON, basal diet; ANTI, basal diet + antibiotics; ESI, basal diet + EOs + SC + IMO. EOs, essential oils. SC, *Saccharomyces cerevisiae*. IMO, isomaltooligosaccharides. Data are presented as mean ± SEM. *** indicate *P *< 0.001.

Figure 2 shows jejunal chyme pH did not differ by diet (*P *> 0.05) but was higher on Farm II versus Farm I (*P *< 0.001). Similarly, ORP was diet-independent (*P *> 0.05) but greater on Farm I versus Farm II (*P *< 0.001). Moreover, a crucial diet × farm interaction was identified for jejunal ORP (*P *< 0.01).

### Jejunal morphology

**Figure 3.**
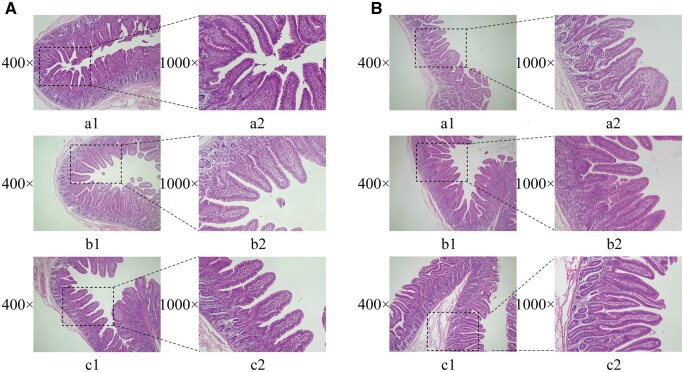
Jejunal tissue morphology in different farms (A: Farm I; B: Farm II). Panels a1, b1, and c1 show jejunal tissue sections under 400× magnification for the following groups: (a1) CON group, basal diet; (b1) ANTI group, basal diet + antibiotics; (c1) ESI group, basal diet + EOs + SC + IMO. EOs, essential oils. SC, *Saccharomyces cerevisiae*. IMO, isomaltooligosaccharides. Panels a2, b2, and c2 show jejunal tissue sections under 1,000× magnification for the same groups.

Histological analysis (Figure 3) demonstrated that both ESI and ANTI treatments observably increased the VH/CD ratio relative to the CON group (*P *< 0.05) (Table 3), while the data indicated that jejunal morphology revealed no significant differences among different farms (*P *> 0.05).

**Table 3.** Effects of ESI on jejunal morphology in weaned piglets across farm environments

Items	Diet treatment^1^			Farm		SEM	P-value		
	CON	ANTI	ESI	I	II		Diet	Farm	Diet × Farm
**Villus height (μm)**	1066.43	1205.46	1119.61	1071.36	1189.64	53.27	0.628	0.330	0.891
**Crypt depth (μm)**	851.82	740.71	752.55	790.07	773.32	26.46	0.235	0.766	0.854
**Villus height/crypt depth**	1.28^b^	1.66^a^	1.55^a^	1.41	1.58	0.057	0.011	0.068	0.862

1 CON, basal diet; ANTI, basal diet + antibiotics; ESI, basal diet + EOs + SC + IMO. EOs, essential oils. SC, Saccharomyces cerevisiae. IMO, isomaltooligosaccharides.

a,b Data in the same row marked with different letters indicate significant differences (P < 0.05), while the same or no letters indicate no significant differences.

### Development-related gene expression in jejunal mucosa

As shown in Table 4, no considerable differences were observed in jejunal mucosal development-related gene expression between the ESI and CON groups (*P *> 0.05). In contrast, the ANTI treatment upregulated *IGF1* and *TGFB1* expression levels compared to the CON group (*P < *0.05). Notably, jejunal *TGFB1* gene expression level was markedly higher on Farm II compared to Farm I and a discernible diet × farm interaction was also detected (*P *< 0.05).

**Table 4.** Effects of ESI on jejunal mucosal development-related gene expression in weaned piglets across farm environments

Items	Diet treatment^1^			Farm		SEM	P-value		
	CON	ANTI	ESI	I	II		Diet	Farm	Diet × Farm
***IGF1***	1.00^b^	1.85^a^	0.50^b^	0.95	1.28	0.193	0.011	0.299	0.712
***IGF1R***	1.00	1.36	0.73	0.78	1.28	0.195	0.411	0.210	0.301
***GCG***	1.00	2.44	1.69	1.56	1.86	0.446	0.514	0.762	0.866
***TGFB1***	1.00^b^	2.92^a^	1.29^b^	0.97^b^	2.51^a^	0.360	0.004	0.002	0.019
***TGFB3***	1.00	1.32	0.47	0.76	1.11	0.184	0.165	0.329	0.334

1 CON group, basal diet; ANTI group, basal diet + antibiotics; ESI group, basal diet + EOs + SC + IMO. EOs, essential oils. SC, *Saccharomyces cerevisiae*. IMO, isomaltooligosaccharides; *IGF1*, insulin-like growth factor-1; *IGF1R*, insulin-like growth factor-1 receptor; *GCG*, glucagon; *TGFB1*, transforming growth factor beta 1; *TGFB3*, transforming growth factor beta 3.

a,b Data in the same row marked with different letters indicate significant differences (*P *< 0.05) between dietary treatment groups or farms. Means sharing the same letter or no letters are not significantly different.

### Serum, liver, and jejunal mucosal immune capacity

As detailed in Table 5, dietary interventions and farm origin differentially modulated immune parameters in weaned piglets. Compared to the CON group, ANTI and ESI treatments noticeably elevated serum IgA levels (*P *< 0.05). Notably, piglets on Farm II had higher serum IgA and IL-1β levels but lower IgG and IL-2 levels than Farm I (*P *< 0.05), with a significant diet × farm interaction in serum IgA level (*P *< 0.05).

**Table 5.** Effects of ESI on serum, liver, and jejunal mucosal immune function of weaned piglets across farm environments

Items	**Diet treatment** ^1^			Farm		SEM	P-value		
	CON	ANTI	ESI	I	II		Diet	Farm	Diet × Farm
**Serum**									
**IgA(μg/mL)**	97.24^b^	137.98^a^	122.49^a^	108.62^b^	129.86^a^	6.569	0.003	0.016	0.016
**IgG(mg/mL)**	50.23	51.68	51.11	61.41^a^	40.60^b^	3.438	0.976	0.002	0.655
**IgM(mg/mL)**	116.67	111.83	113.26	118.82	109.02	3.300	0.856	0.200	0.993
**IL-1β(pg/mL)**	133.83	125.79	131.59	122.93^b^	137.88^a^	2.931	0.303	0.004	0.084
**IL-2(pg/mL)**	112.26	103.27	104.14	110.94^a^	102.18^b^	2.188	0.115	0.030	0.315
**IL-6(pg/mL)**	117.23	120.32	121.11	117.10	122.01	3.324	0.896	0.504	0.376
**TNF-α(pg/mL)**	56.34	62.20	53.66	60.50	54.30	3.889	0.690	0.461	0.371
**IFN-γ(pg/mL)**	127.74	131.9	130.06	130.65	129.15	2.969	0.882	0.828	0.662
**IL-10(pg/mL)**	112.91	119.09	115.62	117.69	114.05	2.923	0.755	0.596	0.928
**Liver**									
**IgA(μg/mgprot)**	48.27^b^	49.03^b^	76.27^a^	85.55^a^	30.16^b^	8.124	<0.001	<0.001	<0.001
**IgG(μg/mgprot)**	45.06	45.63	50.49	53.52^a^	40.60^b^	2.076	0.271	<0.001	0.739
**IgM(μg/mgprot)**	48.89	50.25	55.58	56.68^a^	46.47^b^	2.064	0.245	0.008	0.322
**IL-1β(pg/mgprot)**	59.94	61.85	69.64	68.22^a^	59.41^b^	2.178	0.060	0.016	0.094
**IL-2(pg/mgprot)**	45.70	45.36	49.68	51.66^a^	42.16^b^	1.527	0.208	<0.001	0.849
**IL-6(pg/mgprot)**	53.06	51.72	59.17	60.72^a^	48.58^b^	2.159	0.151	0.002	0.490
**TNF-α(pg/mgprot)**	29.05	28.02	35.48	31.42	30.28	1.686	0.125	0.708	0.129
**IFN-γ(pg/mgprot)**	60.01	60.37	62.99	67.59^a^	54.66^b^	2.070	0.674	<0.001	0.474
**IL-10(pg/mgprot)**	49.66	50.99	57.32	55.06	50.26	1.884	0.251	0.230	0.962

Items	**Diet treatment** ^1^			Farm		SEM	P-value		
	CON	ANTI	ESI	I	II		Diet	Farm	Diet × Farm
**Jejunal mucosa**									
**sIgA(μg/mgprot)**	156.41^b^	233.26^a^	216.97^a^	242.69^a^	233.77^b^	16.47	0.031	0.003	0.167
**IgG(μg/mgprot)**	83.14^b^	126.37^a^	106.40^ab^	124.99^a^	85.62^b^	8.761	0.021	0.003	0.035
**IgM(μg/mgprot)**	98.99^b^	146.07^a^	128.72^ab^	155.01^a^	147.00^b^	11.27	0.048	<0.001	0.092
**IL-1β(pg/mgprot)**	120.02	144.71	121.40	167.36^a^	90.06^b^	12.78	0.407	<0.001	0.105
**IL-2(pg/mgprot)**	101.89	132.36	122.95	142.81^a^	95.33^b^	9.062	0.196	0.004	0.218
**IL-6(pg/mgprot)**	103.02	141.34	129.67	150.06^a^	99.29^b^	10.12	0.084	0.002	0.045
**TNF-α(pg/mgprot)**	73.51	94.07	77.20	94.00^a^	73.51^b^	6.169	0.217	0.024	0.09
**IFN-γ(pg/mgprot)**	122.92^b^	168.73^a^	140.17^ab^	173.66^a^	114.22^b^	11.01	0.039	<0.001	0.049
**IL-10(pg/mgprot)**	116.00^b^	170.18^a^	143.84^ab^	178.04^a^	108.64^b^	12.83	0.044	<0.001	0.074

1 CON group, basal diet; ANTI group, basal diet + antibiotics; ESI group, basal diet + EOs + SC + IMO. EOs, essential oils. SC, *Saccharomyces cerevisiae*. IMO, isomaltooligosaccharides; IgA, immunoglobulin A; sIgA, secretory immunoglobulin A; IgG, immunoglobulin G; IgM, immunoglobulin M; IL-1β, interleukin-1β; IL-2, interleukin-2; IL-6, interleukin-6; TNF-α, tumor necrosis factor alpha; IFN-γ, interferon gamma; IL-10, interleukin-10.

a,b Data in the same row marked with different letters indicate significant differences (*P *< 0.05) between dietary treatment groups or farms. Means sharing the same letter or no letters are not significantly different.

In hepatic tissues, ESI treatment dramatically increased IgA level compared to the CON group (*P *< 0.05). Piglets on Farm I displayed consistently higher levels of IgA, IgG, IgM, IL-1β, IL-2, IL-6, and IFN-γ than Farm II (*P *< 0.05), whereas TNF-α and IL-10 remained comparable across farms (*P *> 0.05). Additionally, a diet × farm interaction was again evident for hepatic IgA (*P *< 0.05).

Jejunal mucosal analysis revealed that ANTI intervention heightened sIgA, IgG, IgM, IFN-γ and IL-10 concentrations and ESI treatment dramatically increased sIgA level compared to the CON group (*P *< 0.05). Piglets on Farm I showed higher sIgA, IgG, IgM, IL-1β, IL-2, IL-6, TNF-α, IFN-γ and IL-10 compared to Farm II (*P *< 0.05). Furthermore, diet × farm interactions were observed for jejunal mucosal IgG, IL-6, and IFN-γ (*P *< 0.05).

### Liver and jejunal mucosal immune-related gene expression

Hepatic expression of pro-inflammatory cytokines (*IL1B*, *IL2*, *IL6*, *IFNG*) was markedly elevated in the ESI group relative to CON and ANTI controls (*P *< 0.05, Table 6). The ANTI group demonstrated selective upregulation of *IL2* and *IFNG* (*P *< 0.01) Moreover, piglets on Farm I displayed consistently lower hepatic expression of *IL1B*, *IL2*, *IL6*, and *IFNG* compared to Farm II (*P *< 0.05), and non-negligible diet × farm interactions were observed for hepatic *IL1B*, *IL2*, and *IL6* expression (*P *< 0.01).

**Table 6.** Effects of ESI on immune-related gene expression in liver and jejunal mucosa of weaned piglets across farm environments

Items	**Diet treatment** ^1^			Farm		SEM	*P*-value		
	CON	ANTI	ESI	I	II		Diet	Farm	Diet × Farm
**Liver**									
***IL1B***	1.00^b^	0.69^b^	1.50^a^	0.45^b^	1.68^a^	0.227	0.002	<0.001	<0.001
***IL2***	1.00^c^	1.65^b^	3.13^a^	1.43^b^	2.43^a^	0.390	<0.001	<0.001	<0.001
***IL6***	1.00^b^	1.24^b^	2.29^a^	1.00^b^	2.02^a^	0.307	<0.001	<0.001	<0.001
***IFNG***	1.00^c^	1.69^b^	4.36^a^	2.16^b^	2.54^a^	0.363	<0.001	0.041	0.221
***IL10***	1.00	1.06	1.11	1.04	1.07	0.023	0.469	0.221	0.846
***PPARG***	1.00	0.81	1.07	0.90	1.02	0.052	0.109	0.233	0.592
**Jejunal mucosa**									
***IL1B***	1.00^b^	0.67^b^	3.88^a^	1.01^b^	2.69^a^	0.506	<0.001	<0.001	<0.001
***IL2***	1.00	0.92	1.13	1.07	0.97	0.043	0.077	0.145	0.038
***IL6***	1.00^c^	1.36^b^	1.92^a^	0.95^b^	1.91^a^	0.189	<0.001	<0.001	<0.001
***IFNG***	1.00^a^	1.12^a^	0.69^b^	1.05^a^	0.82^b^	0.071	<0.001	<0.001	<0.001
***IL10***	1.00^b^	2.64^a^	1.44^b^	1.94^a^	1.44^b^	0.215	<0.001	0.012	0.002
***PPARG***	1.00^c^	1.68^b^	2.37^a^	1.08^b^	2.29^a^	0.258	<0.001	<0.001	<0.001

1 CON group, basal diet; ANTI group, basal diet + antibiotics; ESI group, basal diet + EOs + SC + IMO. EOs, essential oils. SC, *Saccharomyces cerevisiae*. IMO, isomaltooligosaccharides; *IL1B*, interleukin-1 beta; *IL2*, interleukin-2; *IL6*, interleukin-6; *IFNG*, interferon gamma; *IL10*, interleukin-10; *PPARG*, peroxisome proliferators-activated receptors gamma.

a-c Data in the same row marked with different letters indicate significant differences (*P *< 0.05) between dietary treatment groups or farms. Means sharing the same letter or no letters are not significantly different.

In contrast, ESI treatment significantly increased jejunal mucosal *IL1B*, *IL6*, and *PPARG* expression compared to CON group, while *IFNG* gene expression was markedly downregulated (*P *< 0.01). The ANTI group exhibited pronounced upregulation of *IL10* and *PPARG* expression (*P *< 0.01). Farm-specific differences were also evident. Farm I showed significantly lower jejunal mucosal expression of *IL1B*, *IL6*, and *PPARG* but higher *IFNG* and *IL10* mRNA levels compared to Farm II (*P *< 0.05). Furthermore, there were diet × farm interactions in jejunal mucosal gene expression pattern (*P *< 0.05).

## Discussion

Given that EOs, SC, and IMO are promising antibiotic alternatives with distinct action mechanisms, they are likely to exhibit synergistic effects. However, current research primarily focuses on their individual applications, leaving their combined potential in modulating intestinal health and systemic immunity largely unexplored. This study thus evaluated the combined effects of EOs, SC, and IMO on intestinal digestion-absorption capacity and immune responses in weaned piglets across different farming environments. The following discussion interprets these findings with emphasis on their biological significance.

### ESI impacts on piglet intestinal function

Weaning stress often leads to intestinal dysfunction in piglets, particularly affecting the jejunum—the primary site for nutrient digestion and absorption. Structural and functional alterations in jejunum directly compromise piglet growth performance (Tang et al., 2022). Therefore, this study initially quantified jejunal digestive enzyme activities (lipase, amylase and trypsin) in weaned piglets across dietary treatments. Results demonstrated no significant intergroup differences in these parameters. This absence of effect contrasts with several published studies: Zhao et al. (2023) showed that the addition of complex essential oil in diet could improve jejunal trypsin and lipase activities in growing pigs, but without notable change in amylase activity. Similarly, Dong et al. (2024) found that adding a mixture of organic acids and essential oils into broilers’ drinking water significantly increased jejunal lipase activity, but didn’t affect trypsin activity. Furthermore, Zhou et al. (2020) reported enhanced amylase and lipase activities across all intestinal segments with cinnamaldehyde supplementation at 72 and 108 mg kg^−1^ diet in grass carp. These contrasting results may stem from interspecies differences, developmental stages, divergent phytogenic formulations, or distinct administration protocols, warranting further mechanistic investigation.

Suitable intestinal pH can improve the micro-ecological environment, maximize the activity of digestive enzymes, and enhance nutrient absorption (Taubner et al., 2023; Wang et al., 2024). In our study, no notable alteration in jenunal pH level was detected after ESI treatment. Similar findings have been reported in previous studies. Adding different doses of essential oil blend (EOB) (10% thymol, 10% cinnamaldehyde, 10% D-limonene, 7.5% carvacrol, and 62.5% rice bran) or a blend of natural and identical essential oil compounds to the diet wasn’t found to alter jejunal pH value of weaned piglets (Grando et al., 2023; Luise et al., 2023). Consistently, Kashimura et al. (1996) found similar results when 5% IMO supplemented to the diet of rats. Meanwhile, the redox state in the intestine (indicated by ORP), as a balance of oxidation and reduction reactions, is equally crucial for maintaining intestinal physiological functions and metabolic activities (Ruiz-Valdepeñas Montiel et al., 2024). Previous studies have reported elevated intestinal ORP in zebrafish and common carp following a 72-hour exposure to *Aeromonas hydrophila* at 10^8^ CFU/mL (Qi et al., 2023), whereas no significant change in ORP value was observed in our study. This suggests that dietary supplementation with EOs, SC, and IMO had minimal effects on the intestinal luminal environment of weaned piglets.

Due to weaning stress, piglets experience impairment in intestinal morphology and structure, characterized by villous atrophy and rupture (Cao et al., 2022). Intestinal villi are responsible for nutrient absorption. Higher villus height indicates a larger absorption area, while deeper crypts suggest lower cell secretion function and maturity (Uni et al., 1998; Montagne et al., 2003). Numerous studies have shown that adding EOs, SC, IMO, and other additives to animal feed can alleviate adverse effects owing to weaning stress. Notably, Tian and Piao (2019) reported that EOB (4.5% cinnamaldehyde and 13.5% thymol) significantly elevated the jejunal VH/CD ratio in jejunal mucosa. Complementarily, Su et al. (2020) observed that EOB (13.5% thymol and 4.5% cinnamaldehyde) increased jejunal and ileal VH/CD ratios via improving villus architecture and stimulating epithelial cell proliferation. These findings align with our results—a significantly higher jejunal VH/CD ratio in the ESI and ANTI groups, indicating promoted post-weaning jejunal development. This effect is potentially driven by upregulation of intestinal development-related gene expression. Therefore, we further measured the expression levels of *GCG*, *IGF1*, *IGF1R*, *TGFB1*, and *TGFB3* in jejunal mucosa.

The results showed that the ESI treatment did not affect the gene expression related to jejunal mucosa development, whereas marked upregulation of *IGF1* and *TGB1* were observed in the ANTI group. *IGF1* is a key regulator that promotes cell proliferation, differentiation, and protein synthesis of intestinal tissues, playing a crucial role in maintaining the integrity and growth of jejunal mucosa (Michell et al., 1997; Alexander and Carey, 1999). *TGFB1*, on the other hand, is involved in regulating epithelial cell migration, extracellular matrix formation, and tissue repair, which is essential for the structural stability and functional maturation of the jejunal mucosa (Chen et al., 2023). These results suggest that ESI likely enhances the VH/CD ratio through alternative mechanisms distinct from the antibiotic-induced upregulation of *IGF1* and *TGFB1* expression.

### ESI influences on piglet immune responses

Immunoglobulin profiles in weaned piglets serve as key indicators of immunological competence. Among these, secretory IgA (sIgA) in the intestinal mucosa is particularly essential for mediating core mucosal immune functions, such as antigen recognition, pathogen clearance, and maintenance of intestinal barrier integrity (Pietrzak et al., 2020). In our study, ESI treatment promoted a moderate immune response, triggering a significant rise in IgA levels both systemically (in serum and liver) and locally at the mucosal interface (in the jejunum), which underscores its capacity to strengthen integrated immunological barriers. This pattern of systemic and mucosal IgA elevation aligns with the finding of Yang et al. (2023), which reported the increase of serum IgA level in response to *Litsea cubeba* essential oil. Additionally, Yan et al. (2024) demonstrated that co-administration of SC culture and EOs (thymol + cinnamaldehyde) significantly elevated jejunal sIgA level in piglets challenged with enterotoxigenic *Escherichia coli*. Their proposed mechanism—modulation of gut microbiota composition and activation of mucosal immunity, thereby promoting B-cell differentiation into IgA-secreting plasma cells—offers a potential explanation for the sIgA increase observed in the ESI group. In addition, maternal mannan oligosaccharide supplementation was reported to raise sIgA content in piglet jejunal mucosa (Duan et al., 2019), further indicating the immunity-modulating effect of ESI. In contrast, the ANTI group showed broader immunostimulatory effects: elevated serum IgA (systemic) and jejunal sIgA (mucosal), as well as increased jejunal IgG and IgM levels, suggesting a robust enhancement in local immune activity. This comparative analysis suggests that the ESI combination preferentially enhances mucosal immunity (sIgA) and systemic IgA, rather than broadly activating all systemic immunoglobulins like the antibiotic intervention.

A notable finding in the ANTI group was the increase in jejunal mucosal levels of IFN-γ and IL-10, despite no corresponding upregulation of *IFNG* mRNA. This discrepancy suggests post-transcriptional regulation of IFN-γ, possibly involving mechanisms such as mRNA stability, translational efficiency, or protein turnover (Piccirillo et al., 2014). In the ESI group, a more complex regulatory pattern was observed: hepatic *IFNG* expression was upregulated, while jejunal mucosal *IFNG* expression was suppressed, highlighting the tissue-specific mechanisms governing IFN-γ responses. Moreover, the marked upregulation of pro-inflammatory cytokine genes (e.g., *IL1B*, *IL6*) in the ESI group was not accompanied by changes in their protein levels. This dissociation between transcript and protein levels may reflect the operation of post-transcriptional control mechanisms, such as microRNA-mediated repression, regulatory RNA-binding proteins, or feedback loops that fine-tune inflammatory outputs to prevent pathological inflammation (Liu et al., 2016). Therefore, the dissociation between transcript and protein levels warrants further investigation into the potential post-transcriptional immunoregulatory mechanisms of ESI. Understanding how ESI achieves immune activation without concomitant protein production may reveal novel strategies for fine-tuning inflammatory responses.

As a key upstream regulator, peroxisome proliferator-activated receptor gamma (PPAR-γ, encoded by *PPARG*) generally suppresses inflammatory responses by modulating cytokine expression and inhibiting pro-inflammatory signaling pathways, such as NF-κB (Hernandez-Quiles et al., 2021). In our study, both ANTI and ESI treatments upregulated jejunal *PPARG* expression, suggesting that the PPAR-γ pathway may be a target of these interventions. This is consistent with previous reports that resveratrol increases colonic *PPARG* expression in weaned piglets (Gao et al., 2023), and SC restores PPAR-γ-mediated metabolic functions under inflammatory conditions (Zanello et al., 2011). However, the concurrent upregulation of *PPARG* and pro-inflammatory cytokine genes (e.g., IL1B, IL6) appears paradoxical. One plausible explanation is that PPAR-γ activation in specific cellular contexts—such as intestinal epithelial cells versus immune cells—may exert divergent effects (Dubuquoy et al., 2006). Alternatively, the moderate induction of *PPARG* by ESI may be insufficient to fully counteract pro-inflammatory signaling, or it may function as a feedback mechanism to limit excessive inflammation (Straus and Glass, 2007). The dose-dependent effect of essential oils on cytokine responses (Tung et al., 2011; Wei et al., 2017) further complicate this interplay, underscoring the need for future studies to clarify the precise dynamics between PPAR-γ activation and inflammatory cytokine expression.

### Farm environment shapes the efficacy of dietary additives on intestinal function and immune responses in weaned piglets

The randomized block design used in this study not only helped control environmental variability but, more importantly, allowed us to quantify how specific farm conditions modulate the efficacy of dietary interventions. Our results indicate that jejunal digestive enzyme activities and morphological parameters (VH/CD) were not significantly influenced by farm, suggesting that core digestive functions and intestinal structural development remain relatively stable across different rearing environments. However, weaned piglets from Farm I showed significantly higher jejunal redox potential (ORP), reflecting an elevated oxidative stress status. Such a pro-oxidant shift is often linked to inflammatory responses, higher pathogen pressure, or increased physiological stress (Muro et al., 2024). In contrast, piglets from Farm II exhibited significant upregulation of *TGFB1* gene expression in the jejunal mucosa. These observations suggest that the rearing environment at Farm II may be more supportive of jejunal development and immune maturation, whereas the heightened oxidative stress at Farm I may impair normal TGF-β1–mediated regulatory pathways.

Notably, a significant diet × farm interaction was detected for both jejunal ORP and *TGFB1* expression, indicating that the efficacy of dietary additives in modulating intestinal homeostasis is partially dependent on farm-specific conditions. Although environmental parameters such as temperature, humidity, air quality, and stocking density were standardized between the two farms, several key operational differences existed: Farm I used mechanical mixing, warm-air heating, and automated manure removal, whereas Farm II employed manual mixing, heat-lamp heating, and manual manure removal. These distinctions in feeding, thermal regulation, and hygiene management may have contributed to differences in piglet physiological baseline status, particularly in terms of local pathogen pressure and cumulative stress load.

In addition to these operational variations, the observed physiological differences may also reflect “animal source effects”—that is, inherent variations in genetic background, maternal health, sow herd immunity, colostrum composition, and early-life microbial exposure among piglets originating from different farms (Devillers et al., 2007; Guevarra et al., 2019). Such congenital and pre-weaning factors constitute an integral part of the “farm effect” and are not easily eliminated by post-weaning standardization.

The significant diet × farm interactions observed in this study highlight that the efficacy of both ANTI and ESI is modulated by farm-specific conditions, likely mediated through differences in the host’s physiological baseline. Therefore, even under controlled environmental settings, animal source-related factors remain critical in shaping the outcomes of nutritional interventions. Future studies should place greater emphasis on characterizing and incorporating such variables—including genetic background, maternal immune status, and early-life exposures—into both experimental designs and statistical models (Camarinha-Silva et al., 2017). Such efforts will improve the interpretability, reproducibility, and practical applicability of research outcomes, thereby facilitating the development of context-adapted nutritional strategies for diverse swine production systems.

## Conclusion

Essential oils-*Saccharomyces cerevisiae*-isomaltooligosaccharides mixture enhanced intestinal absorptive function by raising the ratio of villus height to crypt depth and boosted immunity through elevating immunoglobulin levels, upregulating hepatic pro-inflammatory cytokine gene expression, and modulating immune gene expression in the jejunal mucosa of weaned ­piglets. These results highlight the potential of the ESI as an antibiotic-alternative dietary supplement that enhances ­gastrointestinal health and overall immunity in weaned piglets.

## Abbreviations:

ANTI group: basal diet + antibiotics
BW: body weight
CD: crypt depth
CON group: basal diet
EOB: essential oil blend
EOs: essential oils
ESI group: basal diet + EOs + SC + IMO
GCG: glucagon
HE: hematoxylin and eosin
IFN-γ: interferon gamma
IgA: immunoglobulin A
IGF1: insulin-like growth factor-1
IGF1R: insulin-like growth factor-1 receptor
IgG: immunoglobulin G
IgM: immunoglobulin M
IL-1β: interleukin-1β
IL-2: interleukin-2
IL-6: interleukin-6
IL-10: interleukin-10
IMO: isomaltooligosaccharides
OD: absorbance
ORP: oxidation-reduction potential
PPARG: peroxisome proliferators-activated receptors gamma
SC: *Saccharomyces cerevisiae*
SEM: standard error of mean
sIgA: secretory immunoglobulin A
TGFB1: transforming growth factor beta 1
TGFB3: transforming growth factor beta 3
TNF-α: tumor necrosis factor alpha
UV: ultraviolet
VH: villus height
VH/CD: villus height/crypt depth
